# Ferrets as Models for Influenza Virus Transmission Studies and Pandemic Risk Assessments

**DOI:** 10.3201/eid2406.172114

**Published:** 2018-06

**Authors:** Jessica A. Belser, Wendy Barclay, Ian Barr, Ron A.M. Fouchier, Ryota Matsuyama, Hiroshi Nishiura, Malik Peiris, Charles J. Russell, Kanta Subbarao, Huachen Zhu, Hui-Ling Yen

**Affiliations:** Centers for Disease Control and Prevention, Atlanta, Georgia, USA (J.A. Belser);; Imperial College, London, UK (W. Barclay); Doherty Institute, Melbourne, Victoria, Australia (I. Barr, K. Subbarao);; Erasmus Medical Center, Rotterdam, the Netherlands (R.A.M. Fouchier);; Hokkaido University, Sapporo, Japan (R. Matsuyama, H. Nishiura);; The University of Hong Kong, Hong Kong, China (M. Peiris, H. Zhu, H.-L. Yen);; St. Jude Children's Research Hospital, Memphis, Tennessee, USA (C.J. Russell)

**Keywords:** ferrets, influenza, viruses, pandemics, risk assessment, transmission

## Abstract

The ferret transmission model is extensively used to assess the pandemic potential of emerging influenza viruses, yet experimental conditions and reported results vary among laboratories. Such variation can be a critical consideration when contextualizing results from independent risk-assessment studies of novel and emerging influenza viruses. To streamline interpretation of data generated in different laboratories, we provide a consensus on experimental parameters that define risk-assessment experiments of influenza virus transmissibility, including disclosure of variables known or suspected to contribute to experimental variability in this model, and advocate adoption of more standardized practices. We also discuss current limitations of the ferret transmission model and highlight continued refinements and advances to this model ongoing in laboratories. Understanding, disclosing, and standardizing the critical parameters of ferret transmission studies will improve the comparability and reproducibility of pandemic influenza risk assessment and increase the statistical power and, perhaps, accuracy of this model.

The susceptibility of ferrets to influenza virus infection has been known for nearly a century. Ferrets and humans share similarities in lung physiology, cellular receptor distribution, and clinical signs of infection, making the ferret an attractive small mammalian model for laboratory study of influenza viruses (*1*). Influenza virus infection in ferrets emulates the severe disease elicited by highly pathogenic avian influenza viruses in humans and the transmissibility of seasonal human influenza viruses via respiratory droplets. Commonly reported experimental setups for the study of influenza virus transmission in ferrets include co-housing influenza virus–infected and uninfected ferrets (previously termed the direct contact model) or physically separating virus-infected and uninfected ferrets (previously termed the respiratory droplet model or the airborne transmission model) (*1*,*2*). In the co-housing design, transmission between ferrets can be mediated by any of the multiple routes that facilitate influenza virus transmission, including direct contact, indirect contact via fomites, or via respiratory droplets (airborne particles with >5 μm aerodynamic diameter) and droplet nuclei (airborne particles with <5 μm aerodynamic diameter). In the physical separation design, direct or indirect contact between donor and recipient ferrets is precluded by separating cages with a side panel or cage walls that permit air exchange between cages but prevent direct contact between ferrets. If the recipient ferret becomes infected with influenza virus, respiratory droplets and droplet nuclei expelled from the donor ferret represent the only possible source of transmission. Many influenza viruses that cause zoonotic infections in humans (e.g., most swine influenza viruses, avian influenza viruses of subtype H5N1 and other subtypes) are generally poorly transmitted between ferrets via respiratory droplets and droplet nuclei, whereas viruses associated with seasonal epidemics or pandemics in humans (e.g., influenza A[H1N1]pdm09 virus, the reconstructed 1918 virus) can be transmitted relatively efficiently (*3*–*7*). As such, the ferret model provides a useful tool for research on influenza virus transmission and pandemic risk assessment. Influenza transmissibility between ferrets is a parameter included in tools for assessing the potential pandemic risk for zoonotic influenza viruses: the Influenza Risk Assessment Tool (*8*) and the World Health Organization Tool for Influenza Pandemic Risk Assessment (*9*).

Recent experimental studies performed by using ferret transmission models have greatly expanded knowledge of influenza virus transmissibility. Among other findings, these studies have identified molecular correlates and determinants of airborne virus spread, differential innate host responses between viruses with distinct transmissible phenotypes, and the role of vaccination and antiviral administration in mitigating virus spread to susceptible contacts (*2*,*10*–*19*). Although the ferret model does not always recapitulate virus transmissibility observed among humans, possibly because of differences in prior exposure history and other unidentified host factors, these studies, in isolation and in tandem with additional laboratory experiments, have improved our ability to perform informative risk assessments of novel and emerging influenza viruses. For example, the limited transmissibility of influenza A(H7N9) viruses via airborne particles in ferrets, in the absence of sustained human-to-human transmission, indicates that the pandemic threat posed by this virus subtype is relatively higher than that of other studied avian influenza viruses; further refinements to the experimental model may help explain why this virus does not currently spread readily between humans.

Despite the growing use of influenza virus transmission studies in the field, there is a wide and often underappreciated heterogeneity among these studies with regard to assessing influenza virus transmissibility via airborne particles in the ferret model. This heterogeneity includes host-specific, virus-specific, and environmental/laboratory-specific variables (Table 1) that are a help and a hindrance for understanding the relative risk for virus transmission with a particular virus strain or subtype (*1*). Robust data can be generated by several independent research groups performing parallel studies with genetically similar but not identical field viruses. Such an approach reduces potential strain-specific or method-specific biases. Conversely, each experimental variable may restrict or complicate direct comparisons of data from different research groups or institutions, depending on each variable’s effect on the transmission outcome, making it impossible to combine data. 

**Table 1:** Examples of heterogeneity in experimental designs among published risk-assessment studies using ferrets as models for influenza virus transmission studies and pandemic risk assessments*

Parameter	Examples of variability
Virus (before ferret introduction)	Seed stock passage history, stock growth matrix, stock titer, wild-type vs. reverse genetics, plaque-purified vs. quasispecies, storage and propagation conditions
Ferret (before virus introduction)	Source/genetic lineage, serostatus, age, sex, weight, neutered or intact status, hormonal treatment (females), anesthetic used, housing conditions
Virus inoculation	Inoculation route, method, dose, and volume; buffer for dilution
Transmission experimental designs	Donor:recipient ratio, number of replicates per containment, caging size and setup, perforation size and exposure area between cages, distance between cages, directional airflow, air changes per hour, temperature and humidity, timing and duration of exposure, frequency and sites for sample collection

*References for individual studies using these conditions are described in (*1*).

Current knowledge about viral, host, and environmental factors that may drive transmission is limited. To facilitate interpretation of data generated in different laboratories, efforts should be made to improve transparency in descriptions of methods and to better differentiate what constitutes a risk-assessment activity versus a research activity. After the Transmission of Respiratory Viruses conference, held June 19–21, 2017, in Hong Kong (*20*), an ancillary workshop was held on June 22, 2017, to discuss this topic. 

Our article serves as a starting point for highlighting potential heterogeneity in ferret transmission experimental designs for future refinement. It is not intended as a policy statement for detailed recommendations on ferret experimental designs, when the effects of many of these variables are not fully understood. We discuss and summarize variables in the ferret transmission model.

## Identifying Variability

Major drivers of variability in studies of influenza virus transmissibility include differences in virus strain, dose, and inoculation route (*21*–*23*). Even when a common virus strain is used, results are potentially affected by the passage condition (e.g., multiplicity of infection), passage history (in embryonated chicken eggs or in mammalian cells), and storage condition of the virus. Risk-assessment studies are particularly challenging because the evaluations are conducted with recently isolated specimens that are not available from a central respository before animal inoculation; variation pertaining to input stock material is possible even when different laboratories have confirmed sequence identity of the same virus undergoing evaluation. Furthermore, several other factors have not been directly demonstrated but are likely to contribute to experimental variation in transmission studies: cage design, airflow direction, air changes per hour, facility-specific temperature and humidity levels, and others (Table 1) (*1*). Beyond these, additional variables, which are largely out of the control of researchers, include ferret suppliers (commercial or hobby and the quantity of ferrets available from each), country-specific animal welfare issues, institutional animal care and use committee guidelines, and pharmaceutical limitations (availability or restriction of anesthetics licensed for use).

Although some factors are considered to be controllable, much of the variability between groups cannot be easily overcome. For example, the absence of commercially available uniform caging for ferret transmission experiments (a reflection of country- and institution-specific size regulations and facilities constraints) represents a parameter that would be difficult to standardize. However, a greater understanding of the relative role and contribution of different variables can improve our ability to better contextualize and interpret results among laboratories. For example, does the virus dose affect transmissibility or otherwise influence detection of transmissible quasispecies in contact ferrets? Gustin et al. previously demonstrated the potential effects of the dose and the route of inoculation (intranasal vs. aerosol) on transmission potential (*22*), suggesting the need to standardize these 2 parameters for risk-assessment studies. Similarly, the use of directional airflow or air changes per hour in ferret housing apparatuses is not always specified in reports of risk-assessment results. Anecdotally, these variables seem to play a role in modulation of virus transmissibility, and they should be examined systematically to ascertain which parameters (including but not limited to those shown in Table 1) would benefit from standardization, where possible, across laboratories in the field. This standardization and interpretation can take place only when all known major drivers of laboratory variability that influence virus transmissibility are described along with the results.

As discussed at the workshop, the participating laboratories analyzed the protocols used for evaluation of influenza virus transmissibility via airborne particles in the ferret model, which highlighted the breadth of experimental designs. It was also clear that all variables that are probable contributors to differential results are not routinely disclosed in peer-reviewed publications or other platforms where results are discussed. Lack of disclosure of all variables can complicate the ease of comparing findings between laboratories, warranting a closer look at the feasibility of using a more comparable study design between multiple laboratory groups for risk-assessment studies. As such, a push toward comprehensive description of specific experimental conditions would aid this effort and would probably be valuable when risk assessments performed in different laboratories are compared.

## Defining Risk Assessment

Beyond the experiment- and facility-based variability we describe, the lack of a standard protocol for the number of experimentally infected animals and the number of recipient ferrets (donor:recipient ratios) included in ferret transmission studies can affect the ability to interpret results among groups and represents a substantial controllable parameter. The ideal standard for risk assessment activities seems to be a 1:1 donor:recipient experimental setup to assess virus transmissibility via the airborne route, where each virus-infected ferret is exposed to only 1 recipient ferret. This design facilitates ease of interpretation of results and provides added rigor from a statistical perspective. This design also restricts exposure to virus-laden particles expelled from each donor ferret to its respective recipient, ensuring that any detected transmission event would have originated from exposure to separate donors. However, because of space limitations, these experiments are often conducted in replicates inside a single physical containment area with a shared ventilation system (i.e., housing multiple pairs of donors and respiratory droplet recipients in separate cages with shared air) while still maintaining a 1:1 donor:recipient ratio. If transmission is mediated by virus-laden particles expelled by donors, increasing the number of donors within a single containment area is likely to increase the concentration of virus-laden particles in the air, thereby increasing the observed transmissibility. Specifically, it is not known whether transmission kinetics would be comparable if 3 independent experiments were performed with 1 donor to 1 recipient (each ferret housed singly) versus 1 experiment with 3 donors and 3 recipients per containment area. Air sampling devices that allow monitoring of the quantities of virus-laden particles in the air throughout the experiment would help refine the experiment outcomes and are likely to become part of these experimental designs in the future.

Further impeding efforts to compare results among laboratories, many experiments include an additional contact ferret co-housed with an experimentally infected ferret to evaluate virus transmissibility in a direct contact setting while still assessing the respiratory droplet transmission to a recipient ferret housed in an adjacent cage. In this design, several ferrets may serve as donors (i.e., virus-inoculated ferrets, co-housed ferrets that became infected as a result of direct/indirect contact, or both). Moreover, the donor and direct contact–infected ferrets are likely to shed virus-laden particles at different times, further complicating the results of the transmission experiments. Ideally, the effects of different experimental designs should be investigated in systematic experiments, and researchers should strive to disclose this information as comprehensively as possible.

During workshop discussions, most researchers agreed that it would be helpful for the field to coalesce around a fixed 1:1 donor:recipient ratio (with or without several discrete pairs inside 1 physical containment area) for risk-assessment transmission experiments. Introduction of direct contact ferrets into the experimental setup would probably extend the amount of time that virus-laden particles can be released in the air. Virus amplification by direct contact ferrets may also lead to virus adaptation and emergence of variants with increased respiratory droplet transmission potential. Applying a 1:1 donor:recipient ratio would increase the consistency of the experimental design under which risk-assessment experiments are conducted across multiple laboratories, differentiating them from broader, more heterogeneous research-based assessments that would include more experimental designs and variables. However, individual laboratories have built up datasets and experience over the years while performing risk-assessment studies with different strains of influenza viruses; thus, adopting a common protocol may be difficult to achieve within a short time.

Although viruses that are readily transmitted by the airborne route will exhibit robust transmission in a direct contact setting, some influenza viruses that are not transmitted efficiently via respiratory droplets are nonetheless transmitted between ferrets placed in direct contact, which facilitates transmission via multiple modes. Studies evaluating virus transmissibility between ferrets placed in direct contact may be influenced by many of the experimental drivers discussed here; when using this model, further contributions to variability are introduced by animal behavior and housing practices. Although scoring for the Centers for Disease Control and Prevention Influenza Risk Assessment Tool includes data derived from the direct contact transmission model in risk assessment, it is not fully clear how to interpret the relative pandemic risk resulting from viruses that transmit in a direct contact setting but not via respiratory droplets. As discussed above, a greater understanding of what confers virus transmissibility in both models will improve our ability to interpret results from more permissive direct contact models with the more stringent respiratory droplet transmissibility. This knowledge will improve our ability to appropriately include and aggregate results from both types of transmission studies in influenza virus risk assessments.

## Limitations of the Ferret Transmission Model

Although the ferret transmission model has greatly improved our understanding of influenza virus transmissibility, there are limits to what this model can contribute to risk assessment and how results are interpreted. In particular, inefficient virus transmission (e.g., when 1 of 3 recipient ferrets becomes productively infected) remains a difficult outcome to understand. It is often unclear whether this event results from genetic changes in the virus during the transmission event, reflects the transmitted infectious dose, or results from other contributing factors; concurrent contextualization of these results with other laboratory paramaters (inclusive of in vivo, in vitro, and aerobiology-based experimentation) can often provide additional insight. Similarly, interpretation of seroconversion in the absence of detectable virus in respiratory secretions or detectable virus in the absence of seroconversion can be difficult. Moving toward a consensus on the implications of inefficient transmission events would be helpful because, currently, efficient and inefficient transmission are not well defined.

Another major limitation of current ferret transmission studies is the small group size, which is driven by cost, size of the animals and their associated housing requirements, and ethical and practical constraints. For this reason, statistical analyses of data from transmission experiments are infrequently performed (*24*,*25*), and repetition of positive-control viruses is not uniformly feasible. Risk assessment studies are often performed with 3–4 replicates of transmission pairs. With this sample size, it is feasible to statistically infer virus transmissibility at the extremes of transmission potential (i.e., virus transmission to 4 of 4 ferrets versus 0 of 4 ferret pairs), but statistical power to compare viruses with intermediate transmissibility (transmissibility to 2 or 3 of 4 pairs) is limited. The opportunity for meta-analyses that combine results from different laboratories could be beneficial, especially for monitoring minor changes in transmission potential of a particular zoonotic virus as it evolves over time. However, meta-analyses can be performed only when experiments use comparable study designs, especially with regard to those parameters known to most dramatically influence virus transmissibility.

## Potential Refinements of the Ferret Transmission Model

Great efforts are being made to reduce the limitations discussed above by using novel and emerging technologies and research-based approaches. For identifying mutations that may have occurred during transmission events, Sanger sequencing has been frequently used. Recently developed technologies (e.g., use of neutral barcodes to individually track influenza viruses in a population) or deep-sequencing approaches have provided, and probably will continue to provide, additional information to aid in the interpretation of inefficient virus transmission events, elucidate transmission bottlenecks, and differentiate between within-group variability and larger differences in experimental setup and design (*26*–*28*). Although incorporating viral genome sequencing in all risk-assessment studies would be beneficial, the inclusion and standardization of these approaches represents a substantial challenge with regard to sample choice for testing (types of samples, dates of sample collection, titers of samples) and institutional restrictions on collection, interpretation, and dissemination of this information.

An additional avenue for improved understanding of virus transmissibility via respiratory droplets are aerobiology-based approaches. These approaches include analysis of the exhaled breath of infected ferrets and the amount and size distribution of virus-containing aerosols released by infected ferrets (*29*–*31*). Although it is unlikely that aerobiology-based information can be incorporated into standard risk-assessment ferret experiments, information gained from these experiments could improve our understanding and interpretation of influenza virus transmissibility in the ferret model, providing additional data about the contributions of different variables to consistency between laboratories for experiments assessing virus transmission.

In vivo ferret transmission studies are not performed in isolation. The incorporation of these data into larger research efforts has greatly expanded our understanding of the complex determinants of influenza virus transmission in mammals. For example, hemagglutinin acid stability and the hemagglutinin–neuraminidase balance have been linked with virus transmissibility via the airborne route in ferrets, as have receptor binding preference, gene constellation, neuraminidase stalk length, and other parameters (*14*–*17*,*28*,*29*). In addition, studies examining the relative effects of environmental temperature and relative humidity on influenza virus stability and transmissibility (underscoring the need to report this information more specifically in published methods sections) will provide needed information pertaining to the seasonality of influenza virus spread in humans (*32*,*33*). Further refinement of the ferret model concurrent with studies using other modeling approaches, including but not limited to in vitro and ex vivo infection models, will continue to support in vivo transmission risk assessments in this species.

## Moving Forward

The plasticity of the ferret model permits a wide range of experimental approaches to assess influenza virus transmissibility. This plasticity represents a great advantage when designing research experiments to evaluate viral, host, environmental, and other factors that contribute to transmission between mammals. However, it might be beneficial for studies conducted primarily for risk-assessment purposes to be performed under conditions as uniform as possible. For example, moving toward a standardized 1:1 donor:recipient ratio in risk-assessment studies would probably enhance the comparability of results found by different research groups and would enable inclusion in meta-analyses (Table 2).

**Table 2:** Features that may be conductive to uniform, reproducible risk-assessment transmission setups when using ferrets as models for for influenza virus transmission studies and pandemic risk assessments*

Property	Rationale	Sample phrasing	Perceived importance
Donor:recipient ratio of 1:1	Improved statistical rigor, potential application for meta-analysis, and interpretation of results. The number of donor:recipient pairs housed inside containment with shared ventilation should be reported.	“Inoculated ferrets (n = 3) were each placed in a separate cage; 24 hours later, naïve ferrets (n = 3) were each placed in a different cage adjacent to an inoculated ferret.”	High
Seronegative ferrets	Prior influenza virus exposure history can be difficult to quantify and control. The methods used for assessing prior exposure should be disclosed.	“Ferrets were serologically negative to currently circulating influenza A (H1N1 and H3N2) and B viruses before challenge, as confirmed by HI assay.”	High
Harmonization of ventilation and environmental conditions	ACH, directional airflow, cage design, humidity/temperature information are reported concurrent with release of results.	“Ferrets were housed for the duration of the experiment in an environmental chamber with HEPA filtration operating at 20 ACH. Airflow velocity was found to be negligible between donor and recipient cages. Ambient temperature (20°–22°C) and relative humidity (40%) were monitored during the experiment.”	High
Uniform definition of efficient transmissibility	Virus titers (with detection limit) and seroconversion are both required to demonstrate robust transmission event.	“Virus transmissibility was confirmed by detection of infectious virus and by seroconversion to homologous virus in recipient ferrets.”	High
Dose, volume, and route of inoculation	Dose of inoculum may affect the transmission kinetics (*22*). A consensus within the risk-assessment group may be beneficial.	“Ferrets were inoculated by the intranasal route with 10^6^ PFU of virus in a volume of 500 μL”	High
Application of air sampling device to determine the size and quantity of virus-laden particles in air	The results may help correlate and refine the transmission phenotype.	“Variables were inclusive of vendor, duration of sampling, specification of collection matrices (buffers, gelatin[s], etc.), specification of virus confirmation via PCR and/or live virus detection, normalization correction of data (if applicable).”	Intermediate

*Discussed at workshop held June 22, 2017, ancillary to Transmission of Respiratory Viruses conference held on June 19–21, 2017, in Hong Kong, China (*20*). ACH, air changes per hour; HEPA, high-efficiency particulate air.

Current knowledge regarding the viral, host, and environmental parameters that drive transmission outcomes is limited. Understanding these parameters would be beneficial for infection control, and future studies should aim to validate these factors empirically. As data regarding the exact role of each of the potential parameters discussed in this article are developed, improved documentation of variables (Table 1) associated with risk assessments would facilitate comparison of data generated across different laboratories. These should include, but are not limited to, stating the stock passage history and storage conditions; dose, volume, and route of inoculation; donor:recipient ratio (and the number of donor:recipient pairs present within a single containment area); and clarity in describing cage setup (coupled with illustrations when possible for easy understanding of the overall experimental conditions), specifying room and cage humidity/temperature, stating airflow directionality when present and air changes per hour, and specifying the condition of sample storage and processing procedures (e.g., whether samples are titered immediately for presence of infectious virus, or whether they are frozen and thawed before use).

Many unanswered questions are relevant for understanding the pandemic risk posed by novel and emerging influenza viruses that lie outside a standardized risk-assessment experimental setup. Some studies that would provide additional valuable information include modulation of distance between cages when assessing virus transmissibility via the airborne route, shortening the duration of exposure between inoculated donors and contact ferrets (*18*,*28*,*34*), and modifying the donor:recipient ferret ratio. Performing these types of experiments according to a standardized risk-assessment evaluation of virus transmissibility could provide valuable contextual information for a more nuanced risk assessment. Distinguishing between risk assessment and research activities will greatly facilitate interpretation and contextualization of data generated by different laboratories.

A useful exercise might be for several research groups to test and compare results of a transmission experiment by using 1 selected virus strain prepared by 1 laboratory. Many practical considerations would need to be discussed, including the particular strain to test and the anticipated transmission phenotype of this virus. Complete standardization of ferret transmission studies conducted worldwide is not possible; however, exercises that seek to examine the relative contributions of laboratory-specific drivers of experimental variability may identify critical parameters or conditions. Similarly, meta-analyses of published data, with included disclosure of the parameters listed in Table 1, could aid in our understanding of the relative contribution of variables involved in respiratory droplet transmission experimentation in ferrets.

Influenza viruses will continue to jump the species barrier and cause human infection and disease. Virus-transmissibility assessments in the ferret model represent one of numerous activities conducted to aid pandemic preparedness efforts in the event that a pandemic virus does emerge in the future. Continued refinement of the ferret model, concurrent with advances in identification of viral, host, and environmental factors that influence transmission risk (*35*), will facilitate assessments of novel and emerging influenza viruses and aid development of better infection control measures.
